# Exploring Molecular Heteroencoders with Latent Space Arithmetic: Atomic Descriptors and Molecular Operators

**DOI:** 10.3390/molecules29163969

**Published:** 2024-08-22

**Authors:** Xinyue Gao, Natalia Baimacheva, Joao Aires-de-Sousa

**Affiliations:** 1 Faculty of Sciences, Université Paris Cité, 75013 Paris, France; 2 Faculty of Chemistry, University of Strasbourg, 4, Blaise Pascal Str., 67081 Strasbourg, France; 3 LAQV and REQUIMTE, Chemistry Department, NOVA School of Science and Technology, Universidade Nova de Lisboa, 2829-516 Caparica, Portugal

**Keywords:** natural language models, atomic descriptors, molecular operators, QSPR

## Abstract

A variational heteroencoder based on recurrent neural networks, trained with SMILES linear notations of molecular structures, was used to derive the following atomic descriptors: delta latent space vectors (DLSVs) obtained from the original SMILES of the whole molecule and the SMILES of the same molecule with the target atom replaced. Different replacements were explored, namely, changing the atomic element, replacement with a character of the model vocabulary not used in the training set, or the removal of the target atom from the SMILES. Unsupervised mapping of the DLSV descriptors with t-distributed stochastic neighbor embedding (t-SNE) revealed a remarkable clustering according to the atomic element, hybridization, atomic type, and aromaticity. Atomic DLSV descriptors were used to train machine learning (ML) models to predict ^19^F NMR chemical shifts. An R^2^ of up to 0.89 and mean absolute errors of up to 5.5 ppm were obtained for an independent test set of 1046 molecules with random forests or a gradient-boosting regressor. Intermediate representations from a Transformer model yielded comparable results. Furthermore, DLSVs were applied as molecular operators in the latent space: the DLSV of a halogenation (H→F substitution) was summed to the LSVs of 4135 new molecules with no fluorine atom and decoded into SMILES, yielding 99% of valid SMILES, with 75% of the SMILES incorporating fluorine and 56% of the structures incorporating fluorine with no other structural change.

## 1. Introduction

Natural language models applied to molecular linear notations have enabled the automatic generation of new molecules (e.g., oriented toward specific chemical spaces [1]), end-to-end property prediction, and unsupervised descriptor generation [2]. Variational autoencoders have been developed with recurrent and convolutional neural networks to transform the SMILES representation of a molecule into a multidimensional vector of continuous values (a latent representation) and transform this vector back into the SMILES notation. The latent representation can be used both for the automatic generation of molecules and as molecular descriptors for quantitative structure–activity relationship (QSAR) tasks [3].

The Transformer language model [4], using a neural network architecture based on attention mechanisms, has been widely adopted for chemical applications via linear notations such as SMILES or InChI. Bjerrum and co-workers [5] presented the Chemformer model for sequence-to-sequence and discriminative chemoinformatics tasks and showed that pre-training a general model to be fine-tuned for downstream tasks improves the performance and convergence speed. Mizuno and co-workers [2] studied how a Transformer model learns chemical structures and partial chemical structures and observed that chirality has a strong influence on the training of the model.

Bjerrum and Sattarov showed that training heteroencoders based on recurrent neural networks (RNNs) to translate between different chemical representations increases the similarity between the latent space distance and the molecular similarity measured with circular fingerprints [6]. Aiming at improving the model’s “understanding” of the input sequence’s “meaning”, Winter and co-workers [7] trained heteroencoders that translate between alternative representations of molecules (SMILES, canonical SMILES, and InChI codes). The models were selected based on the performance of the latent space descriptors in two QSAR tasks. It was observed that the models trained to translate performed better than the models trained to reconstruct codes. An additional prediction task for molecular properties (e.g., logP) during the training also yielded improved molecular descriptors. The best model was based on an RNN and was trained with 72 million molecules to translate SMILES into canonical SMILES. This model performed similarly or better than circular fingerprints and graph-convolution networks in validation QSAR tasks. Other reports have confirmed the high performance of these continuous and data-driven descriptors (CDDDs) in ML studies [2,8,9].

The development of latent space vectors (LSVs) as molecular descriptors is relevant not only for their predictive performance in quantitative structure–property relationship (QSPR) models but also as convenient QSPR-able molecular representations for efficient optimization in the latent space of heteroencoders. Algorithms for multiobjective molecular optimization have integrated the representation of molecules as LSVs, QSAR models, optimization in the latent space, and decoding to generate optimized molecular structures [10].

Molecular heteroencoders have millions of internal parameters (weights), which are learned by training with millions of molecular structures and oriented by observable molecular properties (e.g., biological activities or physicochemical properties). This necessarily contains an immense amount of chemical knowledge that opens the possibility of new explorations with new methods to query these models [2].

Here, we explored a methodology to derive descriptors of atoms in organic molecules using natural language models pre-trained with SMILES strings. The aim of this work was to verify if the derived atomic descriptors (a) have any (chemically significant) relationship with the structural environment of the atom, (b) can be used for QSPR applications and perform better than available descriptors (as has been observed with molecular descriptors in some cases [7]), and (c) can be used as operators in the latent space to transform molecules in a controlled way.

We used an available heteroenconder model [7] to derive the atomic descriptors. The change in the LSV of a molecule when an atom is replaced was explored as a descriptor of that atom, the delta latent space vector (DLSV). The replacement of the target atom is a very convenient way to generate a perturbation focused on an atom within the heteroencoder framework. DLSVs can be used to assess the ability of heteroencoders trained with entire molecules to represent individual substructures, such as atoms in a molecule.

Secondly, we evaluated the ability of the new atomic descriptors to train QSPR models. Although thousands of molecular descriptors have been proposed and can be easily calculated by many available computer programs, atomic descriptors are generally less accessible. They are required for the QSPR/QSAR of properties assigned to individual atoms in molecules [11]. Examples include the prediction of NMR chemical shifts [12,13,14,15,16], partial charges [17,18], local reactivity parameters [19,20], sites of metabolism [21], H-bonding ability [22,23], or the energy of covalent bonds involving hydrogen atoms [24]. Here, we used the DLSVs of atoms to train ML models of Gasteiger partial atomic charges (a *calculated* property) and ^19^F NMR chemical shifts (an *experimental* property).

Finally, we explored the potential of DLSVs as molecular operators in latent space to generate new valid molecules with a desired modification. Latent space arithmetic has been much explored for image processing [25] and has also been used, e.g., to transform the structure of proteins [26] or to propose candidates of ligands and binding proteins in latent space optimizations [27]. Here, the rules for halogenation were defined as Rule = LSV(A) − LSV(B), where A is a molecule with a fluorine atom, and B is the same molecule with the F atom replaced by H. The rule was applied to the LSV of a new molecule to obtain a transformed vector, which was decoded into a SMILES. Results were obtained for a data set with 4135 diverse molecules and were assessed in terms of the percentages of valid SMILES, the incorporation of F atoms, and further modifications in the molecule.

The optimization of chemical structures in the latent space has become a major tool for de novo design, but it is much based on random perturbations in the starting molecule and on arithmetic operations to change molecules in the direction of better-performing ones [10,28]. The establishment of arithmetic operations in the latent space (rules) associated with specific desired transformations opens the way to better-guided algorithms for molecular optimization.

## 2. Results and Discussion

### 2.1. Unsupervised Mapping of Atomic DLSVs with t-SNE

**Figure 1:**
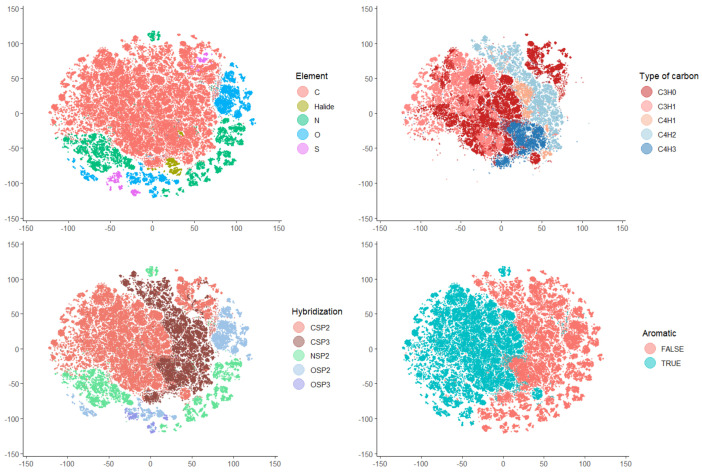
Visualization of selected data points from the t-SNE map of 101,379 atoms based on the DLSV atomic descriptor: atoms of four chemical elements and halides (**upper left**), atoms of five main atomic types based on the number of H and non-H neighbors (**upper right**), atoms of five main hybridization states (**bottom left**), and atoms in aromatic and non-aromatic substructures (**bottom right**).

The ability of atomic DLSVs to represent atoms in molecules was assessed by mapping a data set consisting of >100,000 atoms exhaustively obtained from 5000 diverse molecules. The t-distributed stochastic neighbor embedding (t-SNE) algorithm was used, which can efficiently handle very large data sets. The mapping relied exclusively on the atomic DLSV obtained by the difference between the LSV of the whole molecule and the LSV of the molecule after replacing the target atom with a phosphorus atom. Only after mapping the objects were labels associated with the data points to obtain figures and interactive maps (Figure 1). The interactive maps are available from the github repository associated with this article.

The maps show that the DLSV profiles could discriminate between the atoms of different elements, hybridization, atomic types, and aromaticity. Clustering according to a more detailed environment of atoms (e.g., functional groups) was incipient in some cases, although no clear separation between clusters was observed.

These maps show that DLSVs extract qualitative local information from the LSVs of molecules that can be revealed by unsupervised clustering.

### 2.2. QSPR of Partial Atomic Charges

The ability of DLSV descriptors to train QSPR models was first explored with a *calculated* atomic property as the dependent variable: Gasteiger partial atomic charges. The atomic charge is a property associated with a specific atom in a molecule. Therefore, we investigated whether the atomic descriptors obtained as DLSVs enable machine learning models to predict this property calculated by RDKit from the 2D molecular structure. Differently from the previous experiment, quantitative data were used (in a regression task) to train ML models with supervised learning. The Gasteiger charge is particularly advantageous as a first exploratory property since it is fast to calculate, is easily accessible from the same package used to process the DLSV, and does not depend on the 3D structure.

Four training sets with increasing sizes (100, 500, 1000, and 2000 molecules, each one encompassing the previous) were used to train RF models and tested with the same test set (2182 atoms from 100 molecules). The atoms were represented by their DLSVs obtained by phosphorus replacement. As a benchmark for comparison, RF models were built in the same way with the same training and test sets but using atom-pair fingerprints rooted on the target atom as the atomic descriptors. The fingerprints were calculated by RDKit with rdFingerprintGenerator.GetAtomPairGenerator (fpSize = 512; maxDistance = 3) and the fromAtoms parameter indicating the target atom. The results show that the models trained with DLSV descriptors could learn to predict the atomic charges with only slightly worse accuracy than fingerprints with a small training set (Table 1). The predictions improved with increasing sizes of the training set, more with fingerprints than with DLSVs, to reach R^2^ = 0.923 with the DLSVs. This was the first indication that the DLSV descriptors encode the structural environments of atoms in such a way that the quantitative prediction of properties is possible.

### 2.3. QSPR of ^19^F NMR Chemical Shifts

A second QSPR application was explored to predict the ^19^F NMR chemical shifts. In the data set used, all molecules were associated with only one chemical shift because there was only one fluorine atom per molecule (or a set of equivalent F atoms in CF_2_ or CF_3_ groups), which enabled us to easily replace the target atom in the SMILES to derive the DLSVs. Several alternative replacements of the F atom were investigated, as well as the replacement of all F atoms or only one per molecule. Random forests were first used as the machine learning algorithm and yielded out-of-bag predictions (Table 2). Furthermore, it is even possible, in principle, to use the LSV of the whole original molecule as the independent variables for the prediction of the chemical shift (one chemical shift per molecule can be interpreted as a molecular property instead of an atomic property).

The models trained with the DLSVs yielded significantly better predictions than those with the LSVs of the whole molecule (MAE < 6 ppm vs. 12.4 ppm) and showed that DLSVs can capture relevant information concerning the structural environment of an atom, providing a better framework than LSVs for QSPR models trained with this relatively small data set.

Alternative replacements to generate atomic DLSVs yielded MAE values between 5.8 and 9.6 ppm, replacement with a different halogen performed generally well, and the best results were achieved with the replacement of one F atom with hydrogen and all F atoms with oxygen. Experiments with one F→H replacement and six different initializations of the pseudorandom generator yielded MAE values between 5.83 and 6.00 ppm. The concatenation of two DLSVs obtained with different types of replacement slightly improved the predictions (Table 3). In line with results for other nuclei [29,30], we observed that the Gasteiger partial atomic charges of the F atom and its neighbor C atom were correlated with the ^19^F NMR chemical shifts (R^2^ 0.84 and 0.57, respectively). The concatenation of the Gasteiger charges with the DLSV descriptors improved predictions up to MAE = 4.0 ppm and R^2^ = 0.97.

Other ML algorithms were explored, and the gradient-boosting regressor (GB) performed better than random forests (RFs) (Table 4). The prediction of the independent validation set with these two algorithms and the DLSV oneF→H with and without the Gasteiger charges yielded the results in Table 5. They confirm the results obtained with the out-of-bag estimations on the training set. Restricting the training and testing to CF cases yielded approximately the same results. Despite the evident significance of the models, their accuracy was inferior to that reported by Penner and Vulpetti [14] (MAE = 1.8 ppm) using a rooted fluorine fingerprint descriptor to encode atoms, a variant of the path-based topological torsion count fingerprint using atomic numbers, the number of π electrons, and the number of heavy-atom neighbors to generate 8192 bits. Beyond QSPR models based on atomic fingerprints, the prediction of ^19^F NMR chemical shifts has been reported recently using graph neural networks [31,32], in which molecules enter the neural network directly as graphs with atoms encoded as nodes and bonds as edges. Different data sets were used, achieving MAE values of up to 7.4 ppm [31] and 3.6 ppm [32].

The CDDD heteroencoder was trained with SMILES strings depleted of their stereochemical information. We pre-processed our data set in the same way to calculate the LSVs with the CDDD model. However, the original data set of the ^19^F NMR chemical shifts included stereochemical assignments of the chiral centers in 172 molecules, including 129 cases of molecules with more than one chiral center, which might have an impact on the prediction of the chemical shift. Therefore, additional experiments were performed with a Transformer model [2] trained with a specific focus on the stereochemical information, where 512-feature vectors were generated from intermediate representations and were used similarly to the CDDDs to calculate the delta vectors (oneF→H). These descriptors incorporated stereochemical information because the heteroencoder was trained with chiral molecules represented with stereochemical information in the SMILES strings (namely, via the “@” character), and our data sets were submitted similarly, with assigned stereocenters. To assess the impact of the stereochemical information on the QSPR model’s performance, random forests were independently developed with the DLSVs obtained from SMILES with and without stereochemical information and the same parameters as those for Table 2. The errors obtained without stereochemistry were like those obtained with stereochemistry (with an MAE of 5.9 ppm vs. 6.0 ppm) and similar to the results of the CDDD DLSVs, which indicated no impact of the stereochemical information on this particular QSPR model. Beyond the stereochemical aspect, these results show that atomic descriptors obtained with latent space arithmetic can be successfully derived from different neural network architectures and algorithms.

### 2.4. Molecular Operators

The difference between the LSV of a fluorinated molecule and the LSV for the same molecule with one fluorine atom replaced by hydrogen was calculated for the 997 molecules in the training set of the NMR experiments. These differences were applied as transformation rules to generate new molecules from molecules with no F atom. They were applied to 4135 molecules with no F atoms among the data set of 5000 diverse structures used above for the mapping of atomic descriptors.

The global fluorination rule was derived as the average of all the 997 transformation rules, and it was applied to the 4135 molecules (Table 6). Valid SMILES strings were obtained for 99.42% of the molecules, and 46% of the molecules reacted. H→F replacement was the only change in the structure for 81.55% of the transformed molecules incorporating fluorine, and the average similarity between the starting and the transformed molecules was very high.

Although a single global rule worked remarkably well, we tried to improve the performance by applying a specific rule for each molecule: the rule derived from the most similar molecule. The rationale was that a rule applied to a very dissimilar molecule may result in uncontrolled modifications in the structure. The results (Table 6) show that the main difference was the number of unchanged molecules, which decreased dramatically (from 55% to 20%), while the percentage of structural changes beyond fluorination increased by 7%. Overall, the percentage of molecules resulting in valid SMILES incorporating fluorine as the only modification increased from 34% to 55%.

Nevertheless, a significant number of molecules were unchanged after the rules were applied (20%). Therefore, we tried to apply the same rules multiplied by two. The percentage of valid SMILES decreased to 93%, but only 1.6% of the valid SMILES were unchanged. The percentage of molecules resulting in valid SMILES incorporating fluorine (one or more atoms) as the only modification was 40%. The main difference was in the number of molecules incorporating two atoms of fluorine—48% vs. 0.83%—when the rule was not multiplied.

**Figure 2:**
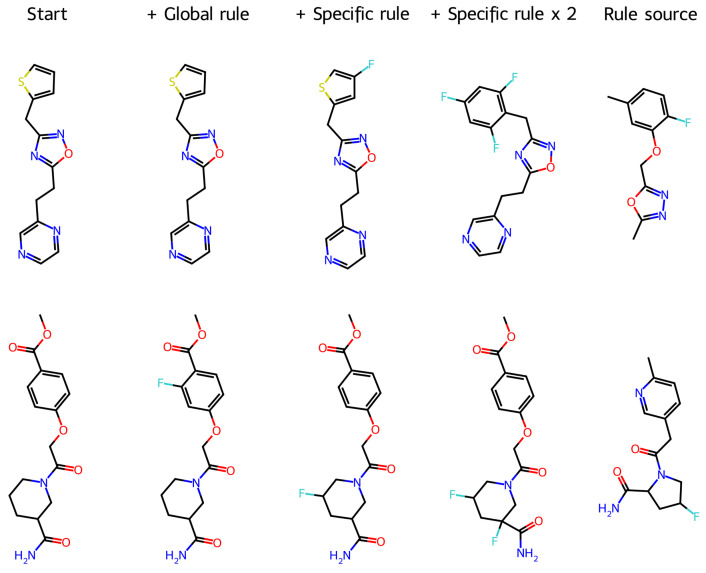
Two examples of molecules transformed by H→F LSV molecular operators (each example includes the starting molecule, the molecule obtained with the global rule, the molecule obtained with the specific rule, the molecule obtained with the specific rule multiplied by 2, and the most similar molecule that was used to build the specific rule).

Figure 2 illustrates the transformation of molecules by the three variants of the rules. In the first example, while the global rule (an average of 997 rules) did not change the structure, the rule obtained from the most similar molecule introduced an F atom in the thiophene ring and made no other change (Tanimoto similarity = 0.91). Application of the same rule multiplied by two introduced three F atoms and transformed the thiophene ring into a substituted benzene ring (similarity = 0.62) inspired by the rule source. Differently, in the second example, all rules introduced one or two F atoms and made no other modification; the global rule replaced an aromatic H atom (similarity = 0.78); the specific rule replaced an H atom of the aliphatic ring (similarity = 0.85); and the specific rule multiplied by two replaced two H atoms of the aliphatic ring (similarity = 0.75). Similarly, the rule source had a fluorine atom in an aliphatic ring. More examples can be found in the Supplementary Materials. As far as the aim of the rules is to introduce fluorine atoms, all these operations may be useful, particularly when the F replacement is the only change. These results suggest that the rules can be built oriented toward more specific transformations, e.g., replacement in an aromatic or aliphatic ring.

The rules to generate F-substituted versions of molecules are presented here as a proof-of-concept case, where transformations can be clearly defined, inspected, and assessed. Furthermore, experimenting with fluorinated analogs is a well-established strategy for drug discovery. DLSV rules enable such a strategy in computational optimizations without decoding into SMILES.

## 3. Methods

### 3.1. Data Sets and Processing of Molecular Structures

A data set of diverse molecules prepared by a random selection of 5000 molecules from the MOSES test_scaffold data sets [33] was used to generate DLSV atomic descriptors for all atoms. Replacement by P on every non-hydrogen atom followed by duplicate removal yielded 101,379 atoms that were mapped with t-SNE. This data set was also used for the QSPR of partial charges and the test set of the halogenation rules.

The data set of ^19^F NMR chemical shifts was obtained from the Local Environment of Fluorine (LEF) QM Tools [14]. It consisted of a training set with 997 molecules and a validation set of 1046 molecules. All molecules had one fluorine atom or a set of NMR-equivalent fluorine atoms (CF_2_ or CF_3_) and the associated NMR chemical shift. The same training and validation sets were used.

The RDKit software version 2020.09.1.0 was used to standardize the molecules, convert between chemical formats, calculate fingerprints, replace atoms, calculate Gasteiger partial charges, perform substructure searches, compare the molecules, and draw, following the package documentation at http://www.rdkit.org (URL accessed on 19 June 2024).

### 3.2. Generation of Delta Latent Space Vectors (DLSVs) of Atoms

**Figure 3:**
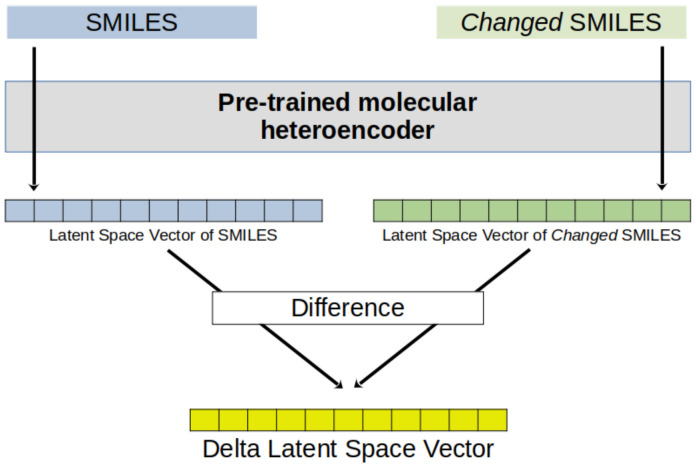
General procedure to obtain delta latent space vectors (DLSVs) from the SMILES string of a molecule and its changed SMILES (SMILES perturbation).

The 512-dimensional latent space vector of each molecule was obtained from its SMILES string by the pre-trained Continuous and Data-Driven Descriptor (CDDD) model available at https://github.com/jrwnter/cddd (URL accessed on 19 June 2024) [7]. The vector was obtained for a molecule before and after the target atom was replaced by a different element, or the target atom in the SMILES representation was replaced by a different character (changed SMILES). The difference between the two vectors was obtained for further processing as an atomic descriptor. A workflow of the procedure to obtain the DLSVs is shown in Figure 3. Several options were tried for the replacement of the target atom: a different element (always the same in the same experiment) or a character with specific consequences in the model embedding. Replacement by a phosphorous atom was convenient, in general, as the CDDD model is prepared to process P atoms, and our data sets included no P atom to be represented. Specific characters were also used: ‘@’ belongs to the dictionary of characters of CDDDs but was not used in the training set (its use is a way to replace an atom with noise); ‘Y’ is not in the CDDD dictionary, activating no token in the input representation. In the case of the ^19^F NMR data set, the DLSV descriptors were generated by removing the F atom(s) from the SMILES string (implicit replacement with hydrogen atoms) or replacing them with Cl, Br, C, N, O, S, @, or Y.

### 3.3. Generation of Rules for Fluorination

The rules for fluorination were derived from the 997 molecules of the ^19^F NMR training set. A rule was defined as Rule = LSV(A) − LSV(B), where A is a molecule from the data set (with one or more fluorine atoms), and B is the same molecule with one F atom replaced by H. The LSVs were obtained with the CDDD heteroencoder. An average rule (global rule) was also generated as the average of the 997 individual rules. The rules were applied to the subset of 4135 molecules with no fluorine atoms from the 5000-molecule set used for t-SNE mapping. A rule was applied to the LSV of a new molecule (C, with no fluorine atoms) to obtain a transformed vector: LSV(D) = LSV(C) + Rule. The new LSV(D) was decoded into the SMILES D with the CDDD heteroencoder and checked for (a) if it was a valid SMILES, (b) how many F atoms were incorporated, and (c) if the H→F substitution was the only transformation. Two different types of experiments were performed, with the average rule applied to all molecules, or with a specific rule applied to each molecule (the rule derived from the most similar molecule). Cosine similarity based on the CDDDs was used to compare the molecules.

### 3.4. Machine Learning Methods

t-distributed stochastic neighbor embedding (t-SNE) was used with the scikit-learn library [34] (version 0.22.2) via the sklearn.manifold.TSNE class to visualize the high-dimensional feature space of 101,379 atoms generated by P replacement on heavy atoms of 5000 molecules. After the calculation of 512 CDDD DLSV descriptors, each entry in our data set also included the SMILES representations, replaced element, atomic type, hybridization state of the replaced atom, and aromaticity. The t-SNE model was fitted to the feature matrix with the following parameters: the number of components (2), automatic learning rate, PCA initialization, and perplexity (30). The visualization of the t-SNE embedding in different features was enabled with the Plotly library to create an interactive scatter plot, where each atom was represented as a point with the t-SNE coordinates.

Random forest (RF) models were built with the scikit-learn library (version 1.2.2) via the sklearn.ensemble.RandomForestRegressor class. The hyperparameters for both the NMR and the atomic charge QSPR experiments were n_estimators = 100, oob_score = True, max_samples = 0.8, and max_features = 0.3. The final models used 400 trees.

Gradient-boosting models were built with the scikit-learn library (version 1.2.2) via the sklearn.ensemble.GradientBoostingRegressor class. The hyperparameters were optimized with the sklearn.model_selection.GridSearchCV class, which performs an exhaustive search across specified parameter values using cross-validation. The optimal parameters were as follows: the number of boosting stages to perform (400), the maximum depth of the individual decision trees (4), the maximum fraction of features to consider when looking for the best split (0.7), the minimum number of samples required to split an internal node (5), the learning rate (0.05), and loss function, “squared_error”.

Multilinear regression models were built with the scikit-learn library (version 1.2.2) via the sklearn.linear_model.LinearRegression class.

Support vector machine (SVM) models were built with the scikit-learn library (version 1.2.2) via the sklearn.svm.SVR class with the kernel function set to ‘linear’ and the other parameters set to the default.

## 4. Conclusions

The delta latent space vectors (DLSVs) obtained with the CDDD model from the SMILES strings of a molecule and the SMILES string of the same molecule with the target atom replaced were explored as descriptors of the atom. The t-SNE mapping of such atomic descriptors with a data set of >100,000 atoms from molecules with C, N, O, S, F, Cl, Br, or H elements revealed clear clustering according to the atomic element, aromaticity, hybridization, and atomic types based on the element and H/non-H neighbors.

The quantitative relationships between the DLSVs and atomic properties (Gasteiger atomic charges and ^19^F NMR chemical shifts) were successful and showed the ability of the new descriptors to make quantitative predictions. The QSPR of the chemical shifts was explored with several machine learning algorithms and a training set of 997 atoms, obtaining predictions for an independent test set with 1046 atoms with an MAE of up to 5.5 ppm and an R^2^ of 0.89. These results were significantly better than the results obtained with the LSVs of the original molecules, demonstrating the chemical significance of DLSVs for the representation of atoms. However, like with the predictions of partial charges, they were inferior to the predictions obtained with the same data set and atomic fingerprints directly derived from the molecular structure. Complementing DLSVs with Gasteiger charges improves the predictions of the chemical shifts to MAE = 3.4 ppm and R^2^ = 0.96. All these results confirm the ability of DLSVs to capture information concerning the structural environment of an atom in a way that correlates with chemical sense and enables QSPR models for NMR chemical shifts. Similar results for the NMR prediction were achieved with the best delta vectors from a Transformer model, which indicates that the methodology is not restricted to a particular neural architecture and algorithm. At the current state of development, the DLSVs could not surpass the performance of conventional fingerprints in the studied QSPR applications.

Meanwhile, latent space arithmetic was successfully applied with the CDDD model to derive rules for halogenation reactions. The application of a single average rule to a data set of 4135 molecules yielded 99.4% of valid SMILES, 42% of which incorporated the F atom (F substitution was the only transformation in 82% of these cases). The application of a specific rule to each molecule, based on the similarity with the rule reactant, increased the number of valid SMILES incorporating an F but also the percentage of molecules with more transformations than the F substitution. Multiplying the rules by two reduced the number of valid SMILES to 93% and increased the number of F-substituted valid SMILES, while further structural transformation became more frequent. This proves that DLSVs can provide efficient rules for guided molecular transformations in the latent space of molecular heteroencoders.

## Figures and Tables

**Table 1:** Random forest prediction of Gasteiger partial charges from DLSVs or atom-pair fingerprints.

Training Set Size (Atoms)	DLSV (MAE|RMSE|R^2^)	AP Fingerprint (MAE|RMSE|R^2^)
2223	0.004|0.064|0.849	0.004|0.060|0.868
10,898	0.003|0.053|0.895	0.001|0.038|0.946
21,844	0.002|0.049|0.911	0.001|0.033|0.960
43,768	0.002|0.046|0.923	0.001|0.030|0.968

**Table 2:** Random forest prediction of ^19^F NMR chemical shifts with DLSVs generated with alternative F atom replacements.

Type of Replacement	MAE (ppm)	RMSE (ppm)	R^2^
All F→H	7.842	12.542	0.848
All F→P	6.896	9.951	0.905
All F→Cl	6.590	9.599	0.911
All F→Br	6.248	9.380	0.915
All F→C	9.221	14.086	0.809
All F→N	7.159	10.507	0.894
All F→O	5.758	8.281	0.934
All F→S	6.842	9.741	0.907
All F→@	8.513	12.937	0.839
All F→Y	8.312	12.585	0.848
One F→H	5.923	9.880	0.906
One F→P	7.673	10.770	0.888
One F→Cl	6.395	9.383	0.915
One F→Br	6.378	9.378	0.915
One F→C	9.611	13.773	0.817
One F→N	8.544	12.172	0.857
One F→O	6.372	9.051	0.921
One F→S	7.437	10.306	0.898
One F→@	8.653	13.213	0.832
One F→Y	8.348	12.569	0.848

**Table 3:** Random forest prediction of ^19^F NMR chemical shifts with alternative/additional descriptors.

Descriptors	MAE (ppm)	RMSE (ppm)	R^2^
LSV original molecule	12.43	18.383	0.675
DLSV AllF→O|OneF→H	5.316	7.956	0.939
DLSV OneF→H|Charges	4.261	6.658	0.957
DLSV AllF→O|Charges	4.188	5.913	0.966
DLSV OneF→H|AllF→O|Charges	4.049	5.945	0.966

**Table 4:** Prediction of ^19^F NMR chemical shifts (ppm) with alternative ML algorithms (descriptors: DLSV One F→H; train/test set split: 8:2; predictions for the test set).

ML Algorithm	MAE (ppm)	RMSE (ppm)	R^2^
GradientBoostingRegressor	3.767	5.490	0.971
MultiLinearRegressor	7.287	9.866	0.907
SupportVectorRegressor	6.094	9.059	0.922
RandomForestRegressor	4.829	7.379	0.948

**Table 5:** Prediction of ^19^F NMR chemical shifts (ppm) for an independent validation set.

Descriptors/Algorithm	MAE (ppm)	RMSE (ppm)	R^2^
DLSV OneF→H|Charges/RF	3.944	6.164	0.95
DLSV OneF→H|Charges/GB	3.526	5.427	0.961
DLSV OneF→H/RF	5.594	9.049	0.892
DLSV OneF→H/GB	5.468	9.793	0.874

**Table 6:** Transformation of molecules by LSV H→F operations.

Rule	% Valid SMILES	% Unchanged	% SMILES W/F ^1^	% Exclusively H→F ^2^	Average Similarity between Start/End Molecules ^3^
Average rule	99.42	55.17	0 F: 5.91 1 F: 93.6 2 F: 0.49	81.55	0.82
Rule of the most similar	98.86	20.23	0 F: 6.13 1 F: 93.0 2 F: 0.83 3 F: 0.03	74.29	0.83
Rule of the most similar × 2	92.94	1.64	0 F: 4.66 1 F: 42.5 2 F: 48.4 3 F: 4.34 4 F: 0.13	46.28	0.74

^1^ Among the valid changed SMILES. ^2^ Among the valid changed SMILES incorporating at least one F atom. ^3^ Average of Tanimoto coefficients between the (RDKit default) fingerprints of the starting molecule and the end molecule, not including the unchanged molecules.

## Data Availability

The script to generate DLSVs with the CDDD model and the whole t-SNE maps are available at https://github.com/jairesdesousa/dlsv (URL accessed on 19 June 2024). The MOSES data set is available at https://github.com/molecularsets/moses (URL accessed on 19 June 2024). The data set of the ^19^F NMR is available at https://github.com/PatrickPenner/lefshift (URL accessed on 19 June 2024). The CDDD model is available at https://github.com/jrwnter/cddd (URL accessed on 19 June 2024). The heteroencoder Transformer model is available at https://github.com/mizuno-group/ChiralityMisunderstanding (URL accessed on 19 June 2024).

## Supplementary Materials

The following supporting information can be downloaded at https://www.mdpi.com/article/10.3390/molecules29163969/s1. Figure S1: Examples of molecules transformed by H→F LSV molecular operators.
