# Supplementary material for: Exploring Molecular Heteroencoders with Latent Space Arithmetic: Atomic Descriptors and Molecular Operators
Source: Molecules. 2024 Aug 22;29(16):3969. doi: 10.3390/molecules29163969 (PMC11357237; doi:10.3390/molecules29163969)
Supplement: Supplementary file 1 [file molecules-29-03969-s001.zip › molecules-3134905-supplementary.pdf]

Supplementary Material

Exploring Molecular Heteroencoders with Latent Space Arithmetic: Atomic Descriptors and Molecular Operators

Xinyue Gao [1], Natalia Baimacheva [2] and Joao Aires-de-Sousa [3,*]

[1] Faculty of Sciences, Université Paris Cité, 75013 Paris, France

[2] Faculty of Chemistry, University of Strasbourg, 4, Blaise Pascal Str., 67081, Strasbourg, France

[3] LAQV and REQUIMTE, Chemistry Department, NOVA School of Science and Technology, Universidade Nova de Lisboa, 2829-516 Caparica, Portugal

[*] Correspondence: jas@fct.unl.pt

Figure S1: Examples of molecules transformed by H→F LSV molecular operators (each example includes the starting molecule (Start), the molecule obtained with the global rule (+ Global rule), the molecule obtained with the specific rule (+ Specific rule), the molecule obtained with the specific rule multiplied by 2 (+ Specific rule × 2), and the most similar molecule that was used to build the specific rule (Rule source).

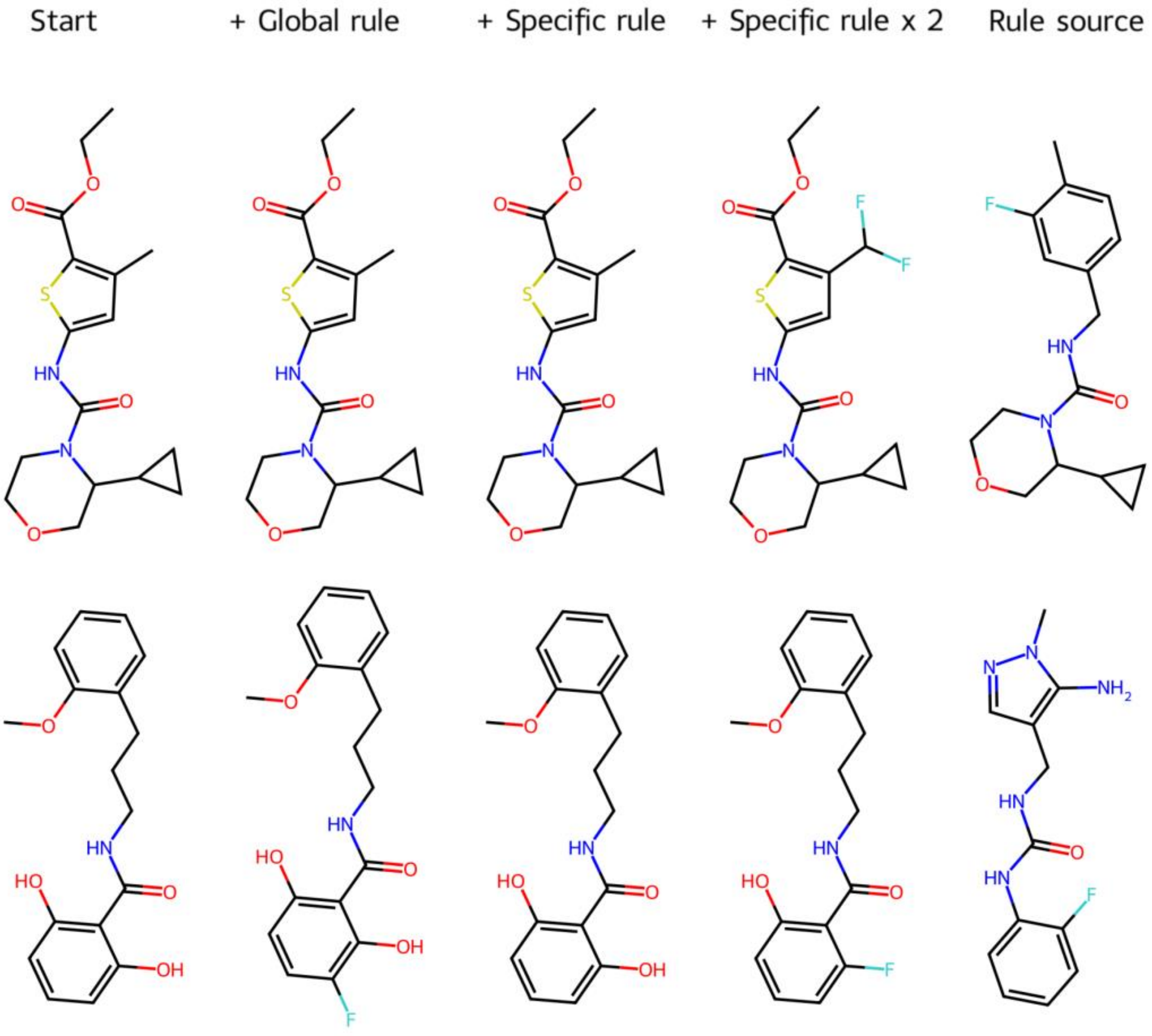

Start	+ Global rule	+ Specific rule	+ Specific rule x 2	Rule source

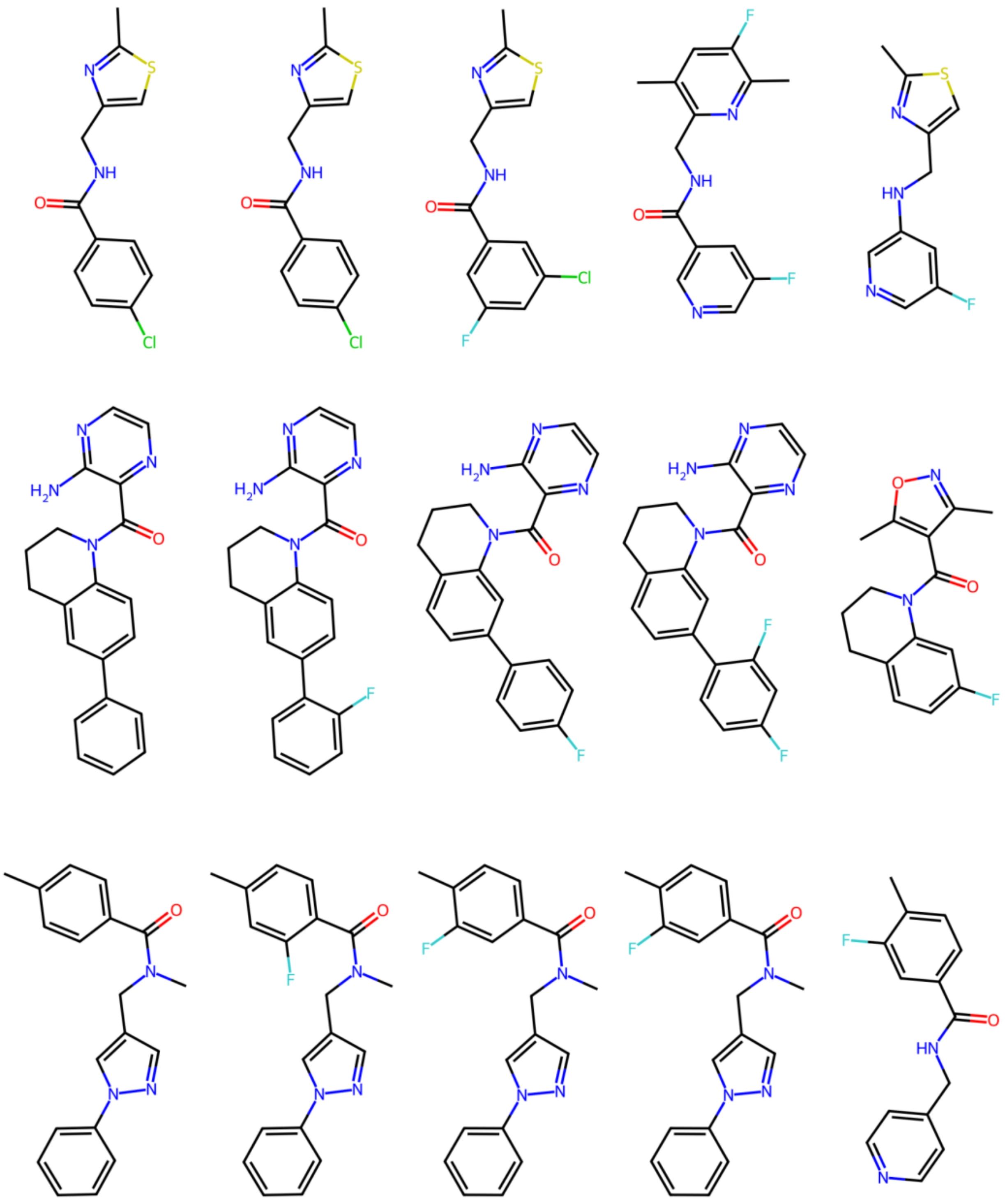

Start | + Global rule | + Specific rule | + Specific rule x 2 | Rule source

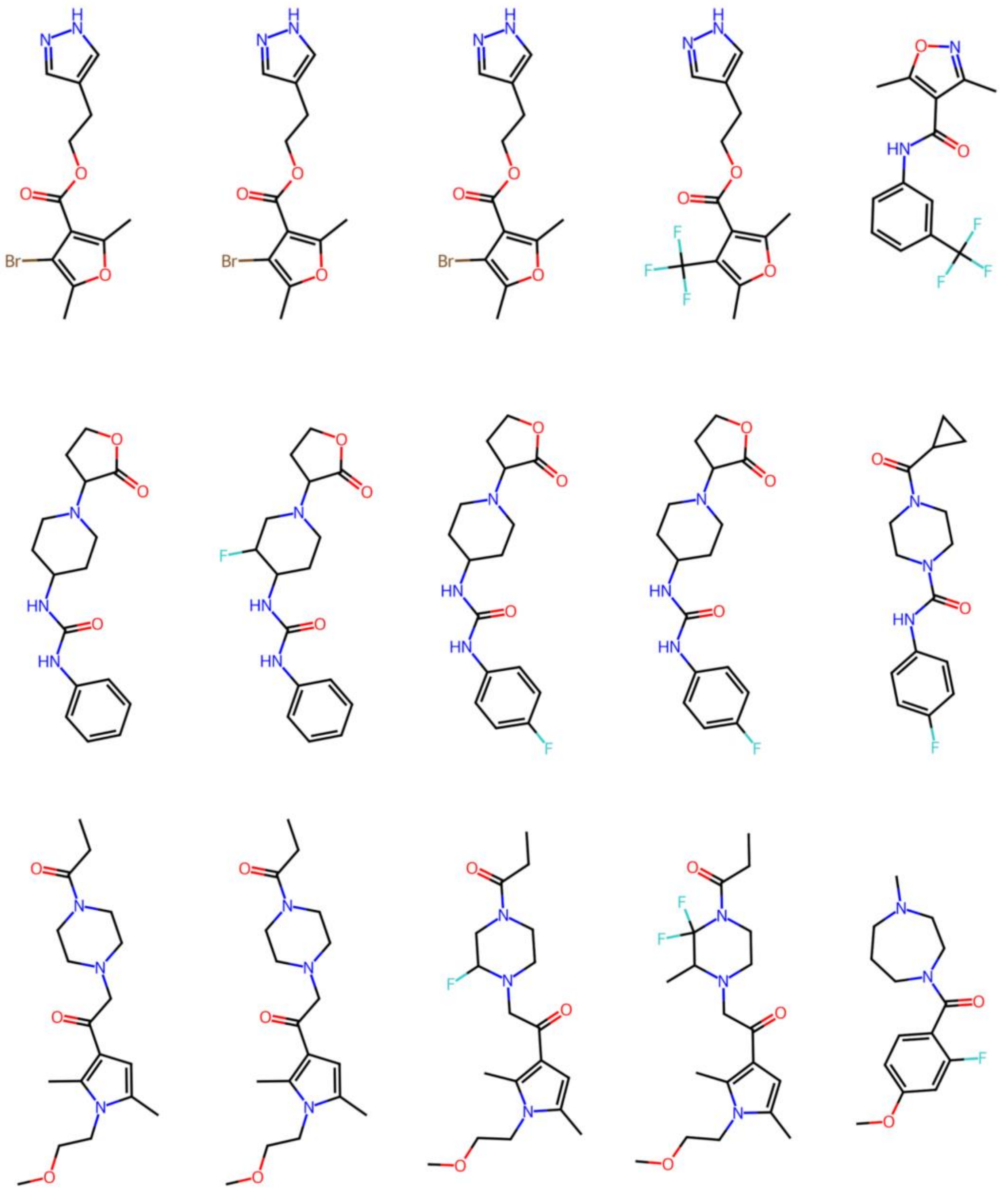

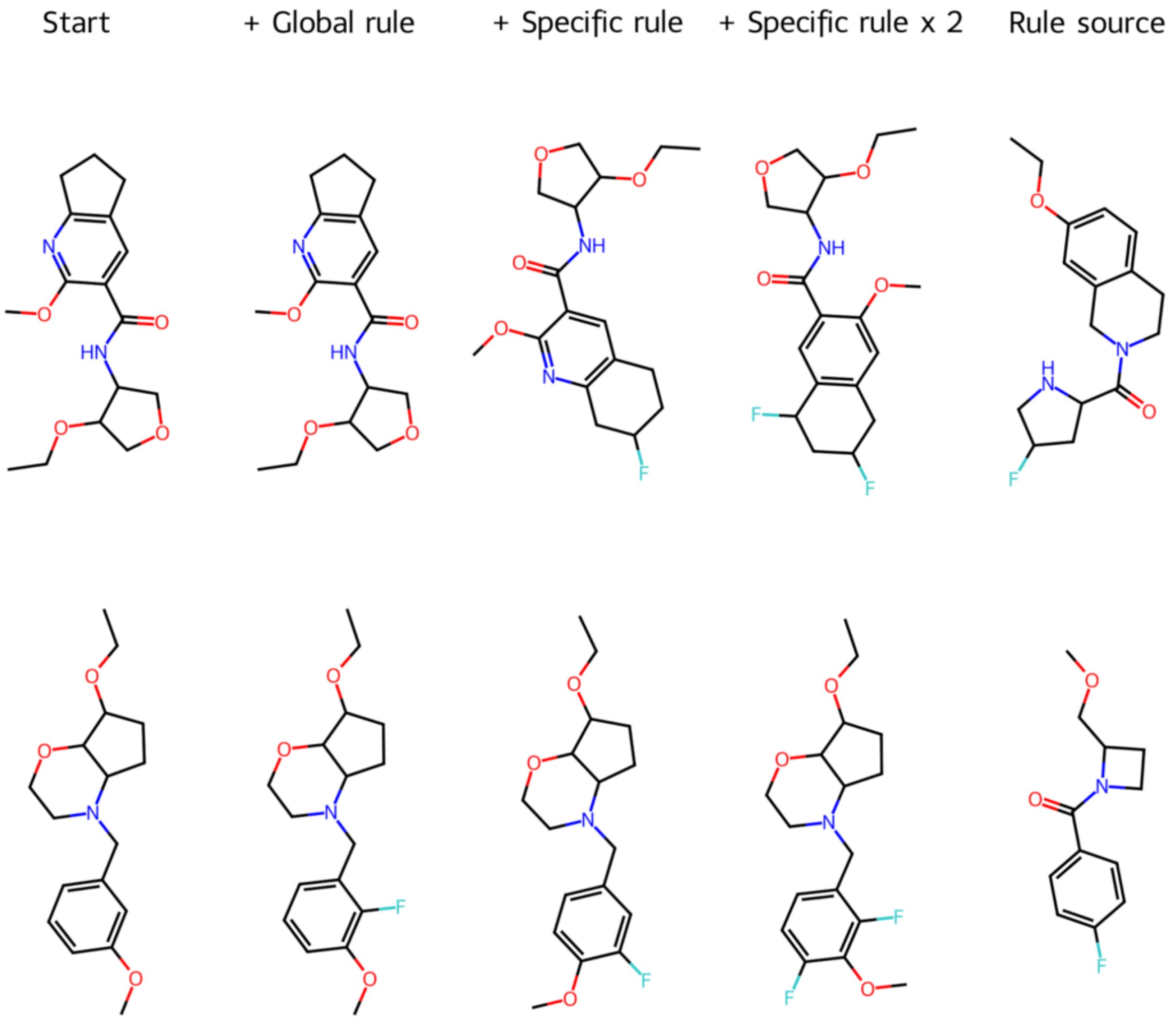
Start
+ Global rule
+ Specific rule
+ Specific rule x 2
Rule source
